# Supplementary material for: Antibody evasion by SARS-CoV-2 Omicron subvariants BA.2.12.1, BA.4 and BA.5
Source: Nature. 2022 Jul 5;608(7923):603–8. doi: 10.1038/s41586-022-05053-w (PMC9385487; doi:10.1038/s41586-022-05053-w)
Supplement: Supplementary file 1 — Reporting Summary. [file 41586_2022_5053_MOESM1_ESM.pdf]

nature portfolio

Corresponding author(s): David D. Ho

Last updated by author(s): Jun 26, 2022

Reporting Summary

Nature Portfolio wishes to improve the reproducibility of the work that we publish. This form provides structure for consistency and transparency in reporting. For further information on Nature Portfolio policies, see our Editorial Policies and the Editorial Policy Checklist.

Statistics

For all statistical analyses, confirm that the following items are present in the figure legend, table legend, main text, or Methods section.

n/a	Confirmed	
☐	☒	The exact sample size (n) for each experimental group/condition, given as a discrete number and unit of measurement
☐	☒	A statement on whether measurements were taken from distinct samples or whether the same sample was measured repeatedly
☐	☒	The statistical test(s) used AND whether they are one- or two-sided<br>*Only common tests should be described solely by name; describe more complex techniques in the Methods section.*
☒	☐	A description of all covariates tested
☒	☐	A description of any assumptions or corrections, such as tests of normality and adjustment for multiple comparisons
☐	☒	A full description of the statistical parameters including central tendency (e.g. means) or other basic estimates (e.g. regression coefficient) AND variation (e.g. standard deviation) or associated estimates of uncertainty (e.g. confidence intervals)
☐	☒	For null hypothesis testing, the test statistic (e.g. F, t, r) with confidence intervals, effect sizes, degrees of freedom and P value noted<br>*Give P values as exact values whenever suitable.*
☒	☐	For Bayesian analysis, information on the choice of priors and Markov chain Monte Carlo settings
☒	☐	For hierarchical and complex designs, identification of the appropriate level for tests and full reporting of outcomes
☒	☐	Estimates of effect sizes (e.g. Cohen's d, Pearson's r), indicating how they were calculated

Our web collection on statistics for biologists contains articles on many of the points above.

Software and code

Policy information about availability of computer code

Data collection	SoftMax Pro 7.0.2 (Molecular Devices, LLC) was used to measure luminescence in the pseudovirus neutralization assays. Biacore T200 biosensor (Cytiva) was used to measure the spike-ACE2 binding affinity.
Data analysis	GraphPad Prism (version 9.2) was used for data visualization and for statistical tests. PISA was used for indetifying antibody-spike interface residues. PyMOL v.2.3.2 was used to perform mutagenesis and to generate structural plots. SPR data were fitted with Biacore T200 Evaluation Software (Version 1.0). The Racmacs package (https://acorg.github.io/Racmacs/, version 1.1.4) was used to generate the antigenic cartography.

For manuscripts utilizing custom algorithms or software that are central to the research but not yet described in published literature, software must be made available to editors and reviewers. We strongly encourage code deposition in a community repository (e.g. GitHub). See the Nature Portfolio guidelines for submitting code & software for further information.

Data

Policy information about availability of data

All manuscripts must include a data availability statement. This statement should provide the following information, where applicable:

- Accession codes, unique identifiers, or web links for publicly available datasets
- A description of any restrictions on data availability
- For clinical datasets or third party data, please ensure that the statement adheres to our policy

All experimental data are provided in the manuscript. Materials used in this study will be available under an appropriated Materials Transfer Agreement. An interactive antigenic map based on the neutralization data of boosted vaccinee sera (Figure 4b) is available online (https://figshare.com/articles/media/OmicronAntigenicMap/19854046). Sequences for Omicron prevalence analysis were downloaded from GISAID (https://www.gisaid.org/). The structures used for analysis in this study are available from PDB under IDs 6ZGE, 7L5B, 6XDG, 7U0N, 7UB0 and 7KMG.

Human research participants

Policy information about studies involving human research participants and Sex and Gender in Research.

Reporting on sex and gender	Sex and gender of the participants in this study are described in detail in the Extended Data Table 2: 30/63 female, 26/63 male, 1/63 intersex, 6/63 unknown sex; 7/63 unknown age, 56/63 22-78 years old.
Population characteristics	A total of 63 individuals were enrolled in this study. Population characteristics for the sera utilized in the pseudovirus neutralization assays are described in the Extended Data Table 2.
Recruitment	Participants volunteered and were enrolled in an observational cohort study at Columbia University Irving Medical Center or at the Hackensack Meridian Center for Discovery and Innovation (CDI) to study the immunological responses to SARS-CoV-2 in individuals who had received COVID-19 vaccines. Self-selection biases may have affected the demographics of the enrolled population, but are not expected to have impacted the results of this study. High titer samples were specifically chosen so that fold-changes in titer could be better determined.
Ethics oversight	All collections were conducted under protocols reviewed and approved by the Institutional Review Board of Columbia University or or the Hackensack Meridian Center for Discovery and Innovation. All of the participants provided written informed consent.

Note that full information on the approval of the study protocol must also be provided in the manuscript.

Field-specific reporting

Please select the one below that is the best fit for your research. If you are not sure, read the appropriate sections before making your selection.

☒ Life sciences ☐ Behavioural & social sciences ☐ Ecological, evolutionary & environmental sciences

For a reference copy of the document with all sections, see nature.com/documents/nr-reporting-summary-flat.pdf

Life sciences study design

All studies must disclose on these points even when the disclosure is negative.

Sample size	No statistical methods were used to predetermine sample size. We used analogous sample sizes as in previous work (e.g. Wang et al 2021, Nature; Liu et al 2022, Nature; Iketani et al 2022, Nature), which we had previously determined to be sufficient sample sizes for comparisons between groups for these experiments. The human research participants (n=63) in this study were characterized in 4 groups, including Boosted (n=16), Non-Omicron infection & vaccination (n=22), BA.1 breakthrough (n=13) and BA.2 breakthrough (n=12).
Data exclusions	No data were excluded.
Replication	The antibody neutralization assays, the serum neutralization assays, the huACE2 inhibition assays were repeated twice independently in technical triplicate with similar results. SPR assays were repeated twice independently with similar results. The results that are shown are representative. All replicates for the neutralization assays and SPR assays are reproducible and successful.
Randomization	As this is an observational study, randomization is not relevant.
Blinding	As this is an observational study, investigators were not blinded.

Reporting for specific materials, systems and methods

We require information from authors about some types of materials, experimental systems and methods used in many studies. Here, indicate whether each material, system or method listed is relevant to your study. If you are not sure if a list item applies to your research, read the appropriate section before selecting a response.

Materials & experimental systems

n/a	Involved in the study
☐	☒ Antibodies
☐	☒ Eukaryotic cell lines
☒	☐ Palaeontology and archaeology
☒	☐ Animals and other organisms
☒	☐ Clinical data
☒	☐ Dual use research of concern

Methods

n/a	Involved in the study
☒	☐ ChIP-seq
☒	☐ Flow cytometry
☒	☐ MRI-based neuroimaging

Antibodies

Antibodies used

All of the antibodies used in this study were produced in our laboratory or provided by other laboratories or companies. 1-20, CAB-A17, LY-CoV555, 2-15, S309, 2-7, LY-CoV1404, ADG-2, DH1047, 10-40, S2X259, 4-18, and 5-7 were expressed and purified in-house as described previously in Liu et al 2020, Nature and in the Methods section of this manuscript. REGN10933, COV2-2196, REGN10987, and COV2-2130 were produced and provided by Regeneron Pharmaceuticals, Brii-196 and Brii-198 were produced and provided by Brii Biosciences, CB6 was produced and provided by Baoshan Zhang and Peter Kwong (NIAID), and ZCB11 was produced and provided by Zhiwei Chen (HKU).

Validation

All of the antibodies have been validated in previous studies by neutralization of SARS-CoV-2. Specifically, 1-20, CB6, Brii-196, REGN10933, COV2-2196, LY-CoV555, 2-15, REGN10987, COV2-2130, LY-CoV1404, Brii-198, S309, 2-7, ADG-2, 10-40, S2X259, 4-18, and 5-7 were tested in Liu et al 2022, Nature, Iketani et al 2022, Nature, or Liu et al 2022, Science Translational Medicine. CAB-A17 and ZCB11 were newly produced and tested prior to use in this study and confirmed to have similar results as that of the original publications (Sheward et al 2022, BioRxiv and Zhou et al 2022, BioRxiv, respectively).

Eukaryotic cell lines

Policy information about cell lines and Sex and Gender in Research

Cell line source(s)

HEK293T cells were obtained from ATCC (Cat #CRL-3216). Vero-E6 cells were obtained from ATCC (Cat #CRL-1586). Expi293 cells were obtained from Thermo Fisher (Cat #A14527).

Authentication

Cells were purchased from authenticated vendors and morphology was confirmed visually before use.

Mycoplasma contamination

cell lines tested mycoplasma negative.

Commonly misidentified lines
(See ICLAC register)

No commonly misidentified cell lines were used in this study.
